# Visuotactile integration modulates motor performance in a perceptual decision-making task

**DOI:** 10.1038/s41598-017-03488-0

**Published:** 2017-06-13

**Authors:** Klaudia Grechuta, Jelena Guga, Giovanni Maffei, Belen Rubio Ballester, Paul F. M. J. Verschure

**Affiliations:** 1 Pompeu Fabra University, Laboratory of Synthetic Perceptive, Emotive and Cognitive Systems, Center of Autonomous Systems and Neurorobotics (SPECS), Barcelona, 08-018 Spain; 2 University of West Bohemia, New Technologies Research Center, Pilsen, 306-14 Czech Republic; 3 ICREA - Institució Catalana de Recerca i Estudis Avançats, Barcelona, 08010 Spain

## Abstract

Body ownership is critically dependent on multimodal integration as for instance revealed in the Rubber Hand Illusion (RHI) and a number of studies which have addressed the neural correlates of the processes underlying this phenomenon. Both experimental and clinical research have shown that the structures underlying body ownership seem to significantly overlap with those of motor control including the parietal and ventral premotor cortices, Temporal Parietal Junction (TPJ) and the insula. This raises the question of whether this structural overlap between body ownership and motor control structures is of any functional significance. Here, we investigate the specific question of whether experimentally induced ownership over a virtual limb can modulate the performance of that limb in a simple sensorimotor task. Using a Virtual reality (VR) environment we modulate body ownership in three experimental conditions with respect to the (in)congruence of stimulus configurations. Our results show that the degree of ownership directly modulates motor performance. This implies that body ownership is not exclusively a perceptual and/or subjective multimodal state but that it is tightly coupled to systems for decision-making and motor control.

## Introduction

In order to successfully act in the world, the brain needs to not only process relevant information about the environment but also store and continuously update the position, rotation and velocity of different parts of the body^1,2^. A simple task, such as intercepting a ball, in practice, requires a number of parallel processes pertaining to both the body and the external world, e.g. postural changes or reaching manipulation^3,4^. Although it has been suggested that much of kinematic control happens outside of perceptual awareness^5^, we can be aware of motion, as opposed to immobility, of different parts of the body even when performing automatic movements^6–8^. This is by virtue of the internal representation of the body^9,10^, conventionally referred to as body ownership^11,12^. Body ownership accounts for the sensory experiences unique to oneself^13,14^ and it results from the integration of somatosensory and vestibular inputs^15^. Similarly to the internal models underlying motor control^16–18^, body ownership is subject to multimodal integration^19,20^ and can be experimentally manipulated^15,21,22^. Interestingly, both experimental and clinical research demonstrate that neural substrates for body ownership and internal models driving fine motor control seem anatomically coupled^6,12,21^.

Temporal plasticity of the body ownership with regards to the respective roles of vision, proprioception, and touch has been studied experimentally in healthy subjects^23^ using the so-called Rubber Hand Illusion (RHI) paradigm. In this experimental setup, the participants were to view a fake hand being stroked in congruence with tactile inputs provided to their real hand, which was visually occluded. The results suggest that the perception of congruent visuotactile stimulation temporarily modulates body ownership resulting in the experience of ownership of the fake hand. This does not occur when conflicting, incongruent visuotactile inputs are provided. Functional Magnetic Resonance (fMRI) and Positron Emission Tomography (PET) studies investigating the neural correlates of sensory integration driving body ownership, demonstrate that RHI correlates with activity in bilateral premotor cortex (PMC), intraparietal sulcus (IPS), sensorimotor cortex, temporo-parietal junction (TPJ) and the right posterior insula^12,21,24^. Indeed, right insular activity had already been reported in the processing^25^, attribution^26^ and recognition of the self^27^ as well as the experience of agency^28^. In a later study, Gentile and colleagues^29^ further validated the multisensory integration hypothesis for bodily self-attribution by comparing the properties of regions which are active during visuotactile unisensory and multisensory stimulations of both real and fake hands using fMRI. In both conditions, the authors found activity in the premotor cortices, the insula and subcortical regions, including the right cerebellum and the left thalamus. Coherent with previous literature, these results suggest that underlying the experience of ownership is a set of regions involved in the recognition of self, such as the insula and TPJ; and motor planning, premotor cortices. Tsakiris and colleagues^20^ showed that the right TPJ correlates with the ability to distinguish self-related events from those generated by the outside world suggesting that it establishes a frame of reference for ownership. Taken together, a number of experimental studies support the notion that body ownership is derived from multisensory integration^21–23^ which correlates with activity in brain structures pertaining to both sensory processing and motor control.

Results from clinical studies investigating pathologies characterized by disturbances of body ownership have provided further evidence for the overlap between sensory and motor areas in body ownership. Somatoparaphrenia, the denial of ownership of a limb or an entire body side, is a consequence of lesions in the right Temporo-Parietal Junction (rTPJ), insula and subcortical regions including the basal ganglia and cerebellum^30^. Furthermore, anosognosia, or the denial of a diagnosed post-stroke motor or sensory impairment, often follows damage to the insula (hyperacute stages), premotor cortex, cingulate gyrus, and TPJ (subacute stages)^6,31,32^. Interestingly, in case of these acquired neurological pathologies, body ownership disorders are often accompanied by contralesional hemiparesis which might disturb all elements of motor control including decision making, planning and action execution^6,30,33,34^, among others^35–39^. Thus the clinical literature supports that disorders of internal representations of the body might be associated with deficits in motor control, which could result from the overlap of the brain structures involved in the processing of ownership and motor control, in particular the bilateral premotor cortices, TPJ as well as the right insula. Following this line of research, the use of efference copies or chorollary discharge (CP) for sensory input filtering has been proposed as a crucial mechanism for the emergence of the subjective experience of motor control and ownership^40^. Indeed, previous research suggests that clinical conditions leading to ownership delusions, such as neuropathic pain and phantom limb, may relate to defective corollary discharge mechanisms^41^. This theory proposes that the accurate virtualization and evaluation of the sensory consequences of self-executed movement may produce the subjective experience of motor control for a specific effector. Little attention has been given, however, to the functional role of this sensorimotor overlap and to the question of whether inducing the experience of ownership may result in a modulation of motor performance.

Here, our goal is to study the relationship between body ownership, decision-making and motor control. In particular, we investigate whether motor performance, in a sensorimotor task, can be modulated by the subjective feeling of ownership over a virtual limb. This modulation is achieved through systematic alteration of the ownership of a virtual arm using the RHI paradigm in Virtual Reality (VR). We devise a protocol to experimentally induce ownership of a virtual hand in healthy subjects, and determine the response times (RTs) in a sensorimotor task where the participants are to deliver rapid motor responses to sensory stimuli (visual or haptic cues) by pressing a button. The degrees of ownership are manipulated across three experimental conditions: congruent visuotactile stimulation (C), incongruent haptic (IH) and incongruent visual (IV) stimulation. Following the RHI paradigm, in the congruent condition, the visuotactile inputs are presented simultaneously, while in the incongruent conditions inputs are delivered asynchronously resulting in visuotactile mismatch. Participants are to respond to the visual and tactile cues in the incongruent visual and the incongruent haptic conditions, respectively. With this design, we on one hand validate previous studies, which found that cross-modal interactions, e.g. haptics and vision, have an effect on the degree of induced ownership, using a VR method^42,43^. Thus we expect that, in the congruent condition, touch is perceived in the location of the virtual hand and the physiological response to an unexpected threatening event presented to the virtual hand is more intense than in IV and IH conditions. Here, we rely on both self-reports^23^ and the Galvanic Skin Responses (GSR) towards a threatening event^44,45^. On the other hand, and most importantly, we analyze whether experimentally induced body ownership driven by visual capture of proprioceptive information modulates motor performance as measured in response times. We expect that in the C motor performance will be faster than both in IV and in IH where the scores will be the lowest. If so, this would suggest a temporal alteration of the internal model that controls overt action possibly deriving form the structural overlap of the brain areas governing sensorimotor processes. Additionally, by deploying two incongruent conditions, where the participants are to rely on either a tactile or a visual cue to execute motor response, we test whether differences in processing of the two sensory stimuli influence the performance on the motor task and whether the sensory weight^29,46^ affects physiological responses towards the threat. Here, we expect that in the IV the motor responses may be faster than in IH condition possibly due to the perceptual prominence of vision over touch^47,48^.

## Methods

### Participants

Thirty six healthy subjects, from the University campus, were recruited for the study, twenty males (mean age 27.85 ± 4.98) and sixteen females (mean age 26.06 ± 9.55). All the participants were right-handed and reported normal or corrected-to-normal vision. Each of the participants was naïve about the purpose of the experiment. Different subjects were randomly assigned to three experimental groups, following a between-subjects design. Such as in ref.^44^, a between-subjects paradigm was chosen to prevent the participants from expecting the threat which could bias the GSR in the subsequent blocks.

The reported experimental procedures with healthy human subjects followed written consents and were in accordance with the established ethical standards, guidelines and regulations. Finally, all the experimental protocols were approved by the University of Pompeu Fabra (Barcelona, Spain).

### Experimental Setup

During the experiment, participants were seated at a table with their right palm placed over a fixed point on the table and the left hand placed in a comfortable position at the left side of the table. The ownership was induced to the right hand while the GSR signal and motor responses were delivered by the left hand. Two Ag–AgCl electrodes were attached to the middle and index fingers of the left hand to record the GSR, and the left thumb was placed on the spacebar to deliver motor responses, which prevented from movement artefacts in the GSR trace.

**Figure 1:**
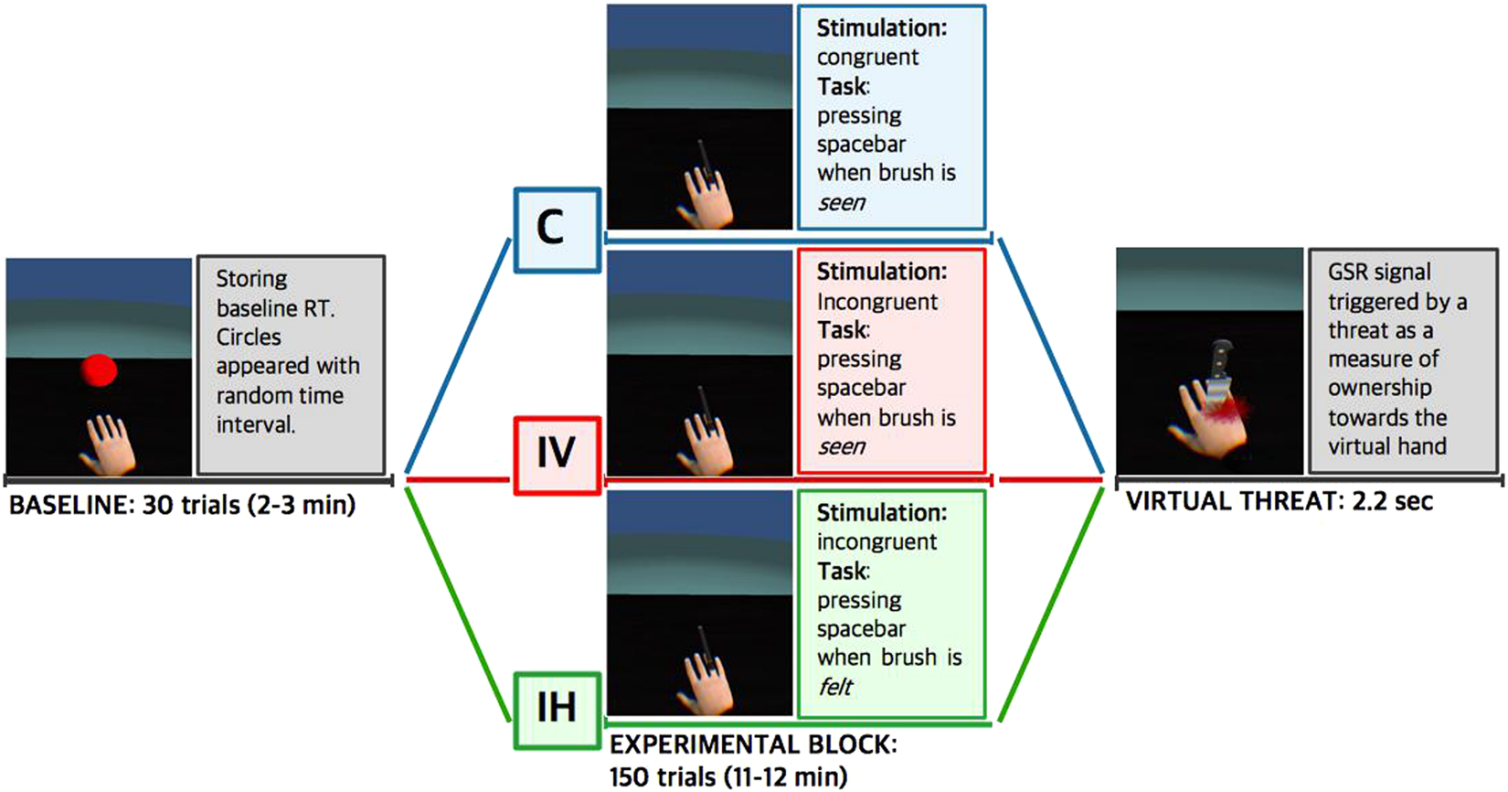
The experimental protocol. BASELINE: baseline block (“spheres”). EXPERIMENTAL BLOCK: the intervention block listing each of the three conditions: C: congruent condition (blue), IV: incongruent visual condition (red), IH: incongruent haptic condition (green). VIRTUAL THREAT: measure of physiological responses to a virtual threat. The same colours are used for every condition throughout the article (C: blue, IV: red and IH: green). Baseline block (A) and the virtual threat (C) were the same for every condition.

The right virtual hand was displayed in front of the participants in a physically credible position^49^, congruent with respect to the real hand (Fig. 1), through a head-mounted display (HMD, Oculus Rift VR DK1, www.oculus.com). Due to contradictory results from previous studies^14,22,44^ which indicated a conflict regarding the physical properties (i.e. size, type or weight) of the two limbs necessary to induce ownership, in the present setup we adopted an anatomically plausible virtual hand (Fig. 1). The tactile stimulation was delivered manually by the experimenter who was seated at the other side of the table, in front of the participant. To fully control for the coincident time onset of the visual (computer generated) and tactile (manually delivered) inputs, the experimenter received precise timing instructions through headphones. For the data analysis, the participants’ responses were time locked between the sensory inputs and motor commands, both stored by the system. A paintbrush was used to perform the stroking, and the length of the visuotactile stimulus for every finger was approximately 1.4 seconds long. Virtual analogue of the real brush was accordingly visualized through the HMD.

Within the virtual scene (Fig. 1), everyone viewed the stimulated virtual analogue of the real right hand which was resting on the table, and the according stimuli in the baseline and intervention blocks. Thus the use of VR allowed us to control for what the participants were exposed to throughout the experiment. In particular, when the Virtual Threat was presented it allowed us to control for unrelated visual factors which could influence the GSR signal. Finally, the present method prevented from possible biases in the performance caused by the presence of the experimenter^50^.

### Experimental Protocol

The study consisted of two experimental blocks (Fig. 1): a baseline block and an intervention block. The first block, or the baseline block (2–3 minutes) was identical for every condition. During this block, the participants were required to provide a motor response as soon as a red sphere appeared in the display in front of them. All the spheres had identical properties and they always appeared in the same position (Fig. 1, BASELINE). The spheres were displayed with random inter stimulus intervals (1–3 seconds) and each exposure lasted 1.4 seconds. This block consisted of 30 trials and served to calculate baseline RTs for every participant. Given the between-subjects design, it allowed us to account for the potential inter-subjects’ variability (i.e. psychophysical differences) and to compare the unbiased motor responses between conditions (i.e. C, IV and IH). The averaged baseline RTs for every participant were later subtracted from the intervention block. Both in the baseline and intervention blocks, the motor responses consisted in pressing the spacebar with the left thumb. Each experimental session in all conditions had an approximate duration of 25 minutes.

The three experimental conditions included C: congruent condition, IV: incongruent visual condition, IH: incongruent haptic condition (Fig. 1), each of which was followed by a threating event. In these intervention blocks, while the visuotactile stimulation was delivered, the participants were asked to provide a motor response as soon as the right index finger was being stroked. This block consisted of 150 trials and the visual and tactile feedbacks were manipulated across three conditions (Fig. 1, EXPERIMENTAL BLOCK). In C, the act of stroking with the brush seen on the screen was congruent with tactile stimulation of the real hand. The real finger and the virtual analogue of the same finger were brushed congruently and the participants were instructed to respond to the visual stimuli (the participants were verbally given the following instruction: “Please, press the spacebar, with your left thumb, as soon as the index finger of the virtual right hand is stroked”). In IV condition, the act of stroking with the brush seen on the screen was incongruent with tactile stimulation of the real hand. The participants viewed a different finger being stroked than the one stroked on the real hand. Here, the participants were instructed to respond only to the visual stimuli (the participants were verbally given the instruction: “Please, press the spacebar, with your left thumb, as soon as you see that the brush strokes the index finger of the right virtual hand”). The IH condition followed the same procedure as the IV, but the participants were asked to respond to haptic stimuli as opposed to visual (“Please, press the spacebar, with your left thumb, as soon as you feel that the brush strokes the index finger of the real right hand”). All the five fingers were brushed in an unpredictable pseudo-randomized sequence, counterbalanced within every session. To account for potential order-effects, we computed a different sequence of strokes for each participant following the same pseudo-random order.

In order to investigate whether the experimentally induced ownership modulates decision-making and motor responses, in every condition, the participants were asked to respond only when the index finger in being stroked. No action was required when the stimulus was provided to other fingers. Furthermore, since the response times can be influenced by instructions emphasizing either speed or accuracy^51^, to prevent errors, in our paradigm, the participants were instructed to provide the response when the stroking began, but the task did not impose a speed limit (i.e. no error notification). With such design, at every stroking event, the participants needed to make perceptual decisions of weather to execute the motor action, or not, depending on the visual or tactile inputs provided, while no speed-accuracy tradeoff was expected. We predicted that in the C condition, with higher ownership, the motor responses will be faster than in both IH and IV. We further hypothesized that the responses in IH might be slower than in IV possibly due to the prominence of vision over touch^29^.

At the end of every intervention block, a virtual knife appeared to serve as a threat to the fake hand (Fig. 1, VIRTUAL THREAT). The knife descended from the top of the screen and into the dorsal part of the virtual hand. The animation gave the impression of the virtual hand being stabbed, which was emphasized by a momentary bout of bleeding emerging from the wound. Both the knife and the blood vanished after less half a second (300 ms.). The whole animation lasted 2.2 seconds in total and the participants were instructed to stay seated, with the HMD on, for another 60 seconds. With this method, we could objectively validate whether synchronous visual and tactile stimulation of the virtual and the real hands can induce the feeling of ownership using the proposed virtual-reality protocol as shown in^44,52,53^. Secondly, comparison of motor performance and ownership between the conditions, allowed us to investigate our primarily goal, namely, whether the modulation of the representation of the body results in faster responses and a better performance on the proposed motor task. Additionally, by comparing the results from the control conditions (IV and IH) we could further assess whether the performance can be affected by attending to different modalities and whether this influences the GSR responses.

### Measures

#### Self-report

After every experiment, the participants completed a questionnaire, which consisted of nine questions, three of which were related to the perceptual experience of ownership, while the remaining six served as controls. Subjects were asked to respond by rating their level of agreement on a 7-point Likert scale (−3: strongly disagree, 3: strongly agree). The questions were adapted from the previous RHI studies^21,23^ to fit the present VR paradigm. The order of the questions was randomized across subjects to avoid order effects. The three questions related to the ownership included: “I had the feeling that I was receiving the touch of the brush in the location of the virtual hand” (Q1), “It seemed as if the touch I felt was caused by the brush that I was seeing on the screen” (Q2), and “I felt as if the virtual hand was my own” (Q3). While the control questions were: “It seemed that my real hand was being displaced towards the left (towards the virtual hand)”, “It seemed that the touch that I was feeling originated in some place in between my own hand and the virtual hand”, “I felt as if my real hand was becoming virtual”, “It seemed (visually) that the virtual hand was being displaced towards the right (towards my real hand)”, “The virtual hand started to look like my own hand in some aspects”, and “I had the sensation of having more than one right hand”.

#### Galvanic Skin Response (GSR)

The Autonomic Nervous System (ANS) is the primary mechanism which regulates involuntarily physiological states, such as arousal produced due to anticipating pain or fear. We used GSR (the electrical conductance of the skin), as a measure of ANS activity to further quantify the experience of ownership over the virtual hand and compare our results with previous studies^44^. We expected that all subjects would show changes in GSRs after the threatening event (VT), but that there would be higher responses in the congruent (C) condition due to the enhanced assimilation of the virtual hand into the perceptual bodily representation.

The GSR was recorded throughout the experiment with two Ag–AgCl electrodes attached to the palmar surface of the index and middle fingers of the participants’ left hand (e-Health Sensor Platform V2.0, Cooking hacks, Zaragoza, Spain) and the data was recorded using an Arduino microcontroller^54^. We measured GSR during the entire experiment, however, we were particularly interested in the GSR responses to the VT displayed at the end of the experiment in every condition (Fig. 1, VIRTUAL THREAT). The timing of the threat event was stored and registered with the GSR and behavioral record for further analysis. In order to compare the GSR responses between the three conditions, we defined a latency onset window of 12 seconds after the stimulus onset. The GSR signal after VT was normalized for every participant by subtracting the mean signal from 12 seconds prior to the stimulus onset.

#### Response Times (RTs)

In the baseline block (“Spheres”), all the participants were asked to provide a motor response (press the spacebar) as soon as a red sphere appeared in front of them. To calculate the baseline and account for individual differences between subjects (i.e. psychophysical inter-subject variability), we stored the RTs for every participant, which we defined as the time interval between the onset of the sphere and motor response. During the intervention block, (Fig. 1, EXPERIMENTAL BLOCK) the RTs were defined as the intervals between the beginning of stroking and motor response. In both blocks, the RTs were used as a measure of perceptual detection and motor performance^29,55^. For the data analysis, we normalized the RTs in the intervention block for every participant by subtracting their mean response time from the baseline block (“spheres”).

## Results

The goal of the present study was first to devise and validate a VR paradigm of the standard RHI protocol^2,21,23^ following two ownership induction methods (i.e. congruent and incongruent). Second, and most importantly, we evaluated the effect of ownership of the virtual hand, as measured by self-reports^23^ and GSR responses to a virtual threat^44^, on motor performance in the proposed task. We expected that ownership might have a modulatory effect on motor performance as measured through RTs such that in C the performance will be better than in both control conditions (IV and IH). Finally, grounded in the theories of the dominance of vision over touch^47^, we further hypothesized that in IV the motor performance can be faster than in IH condition.

Normality test revealed that GSR and RTs data were not normally distributed. Consequently, the statistical analysis followed nonparametric analysis. We used Kruskal-Wallis (KW) tests between conditions, corrected for multiple comparisons and a Mann–Whitney U test to identify differences between groups. A Pearson product-moment correlation coefficient was computed for the subsequent linear correlation analyses.

### Self-reported feeling of ownership

**Figure 2:**
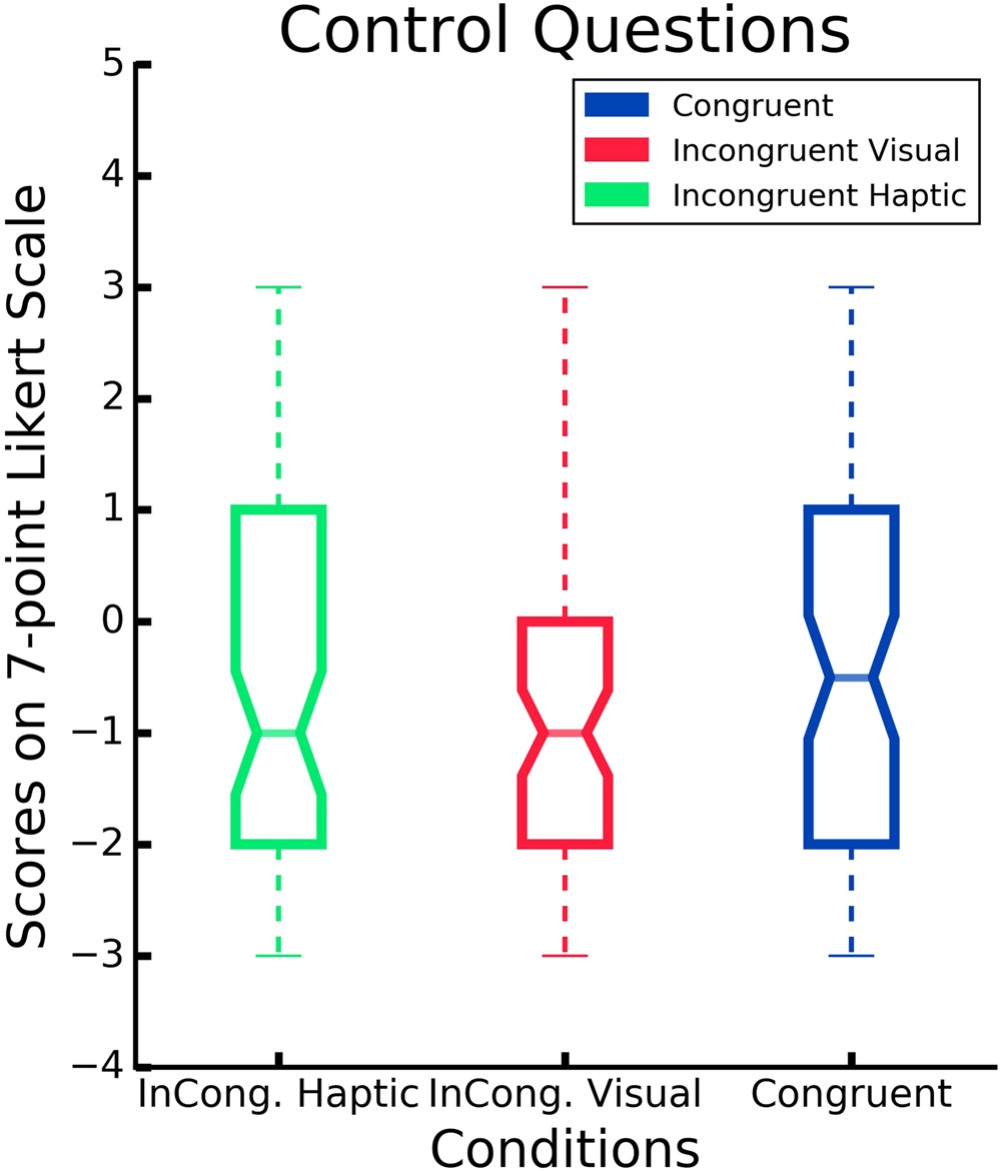
Self-reported experience of ownership. Y-axis: Responses on the 7-point Likert scale ranging from −3 (strongly disagree) to 3 (strongly agree). Scores above 0 indicate a feeling of ownership. Control Questions: mean of the three questions related to the ownership illusion per condition.

**Figure 3:**
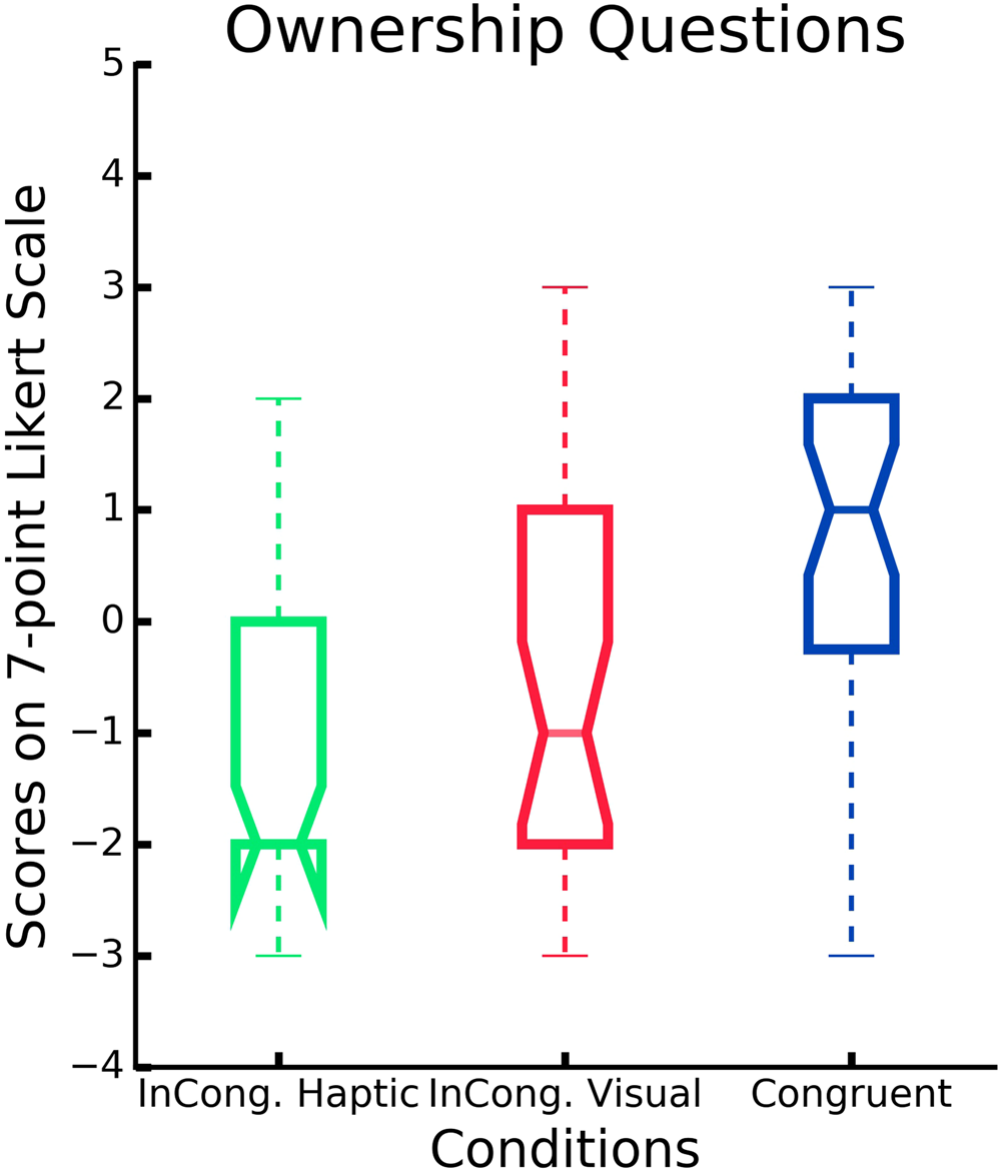
Self-reported experience of ownership. Y-axis: Responses on the 7-point Likert scale ranging from −3 (strongly disagree) to 3 (strongly agree). Scores above 0 indicate a feeling of ownership. Ownership Questions: mean of the six control questions per condition.

After each experimental session, the participants were required to rate the level of perceived ownership. Results show that the congruent visuotactile stimulation of the virtual and real hands (i.e. condition C) enhanced the feeling of ownership, compared to the control conditions IV and IH (*p* = 0.016) (Fig. 3). The mean score across the participants for Q1, Q2 and Q3 in condition C was 0.78 (*SD* = 1.82), −0.76 (*SD* = 1.75) in IV, and −1.0 (*SD* = 1.39) in IH. Kruskal–Wallis (KW) test showed that there was a significant difference between the three conditions (C, IV, IH) for the three ownership questions (*H*(2,36) = 18.71, *p* < 0.001). In particular, we followed the previous finding with a Mann-Whitney U test, which indicated that the scores in condition C were significantly higher than both conditions IV (*Mdn* = −1, *U* = 323, *p* < 0.001), and IH (*Mdn* = −2, *U* = 292.5, *p* < 0.001). No significant difference was found between the control conditions, IV and IH (*U* = 556.5, *p* = 0.32) (Fig. 3). The mean rating across all participants for the six control statements (Q4, Q5, Q6, Q7, Q8, Q9) was −0.36 (*SD* = 2.13) for condition C, −0.75 (*SD* = 1.68) in IV, and −0.61 (*SD* = 1.96) in IH. No difference was found between the conditions in the control questions (*KW*, *H*(2,36) = 0.81, *p* = 0.67) (Fig. 2). Thus self-reported feeling of ownership occurred only in condition C.

### Physiological measures of ownership illusion

**Figure 4:**
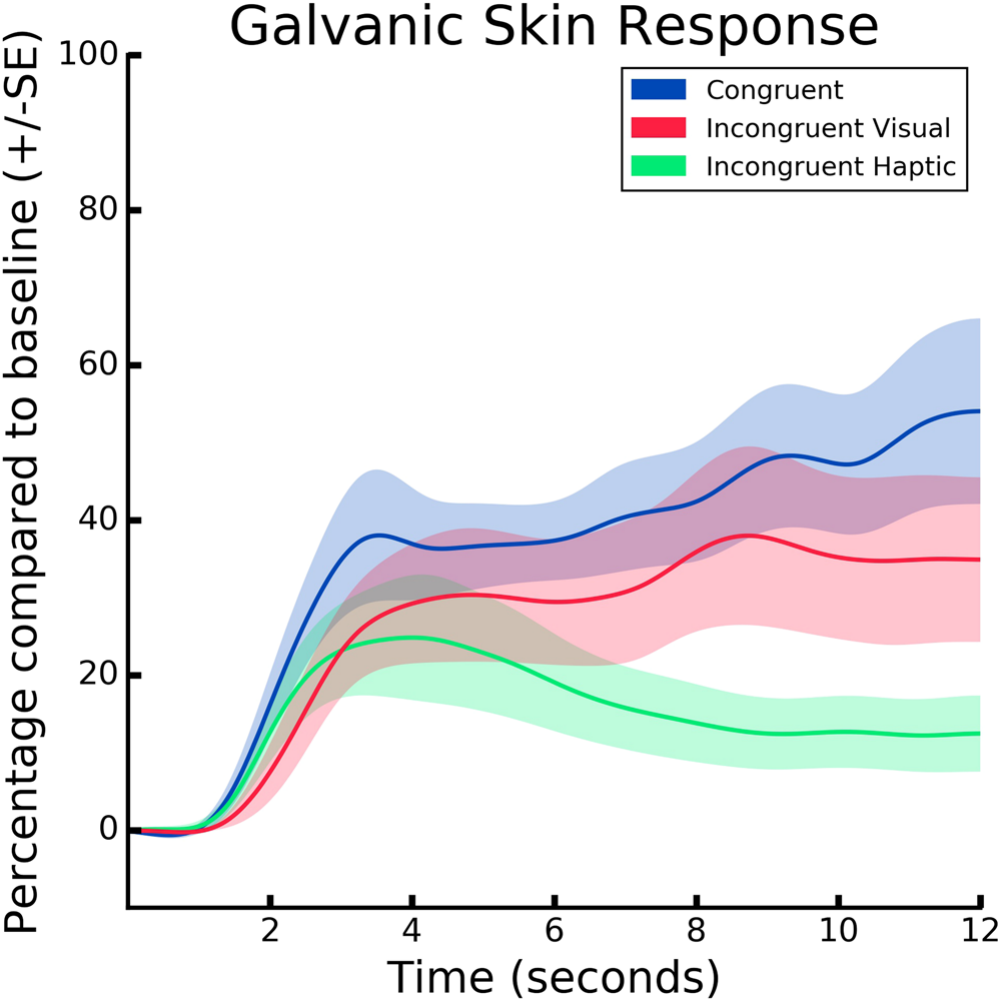
GSR results. The sampling rate for the GSR signal was 60 Hz. Accordingly, the data were run through a low-pass filter with a cut-off frequency of 0.06 Hz. Mean GSR responses per condition for all the participants averaged in a time window of 12 sec. The threatening event happened at time = 0.

**Figure 5:**
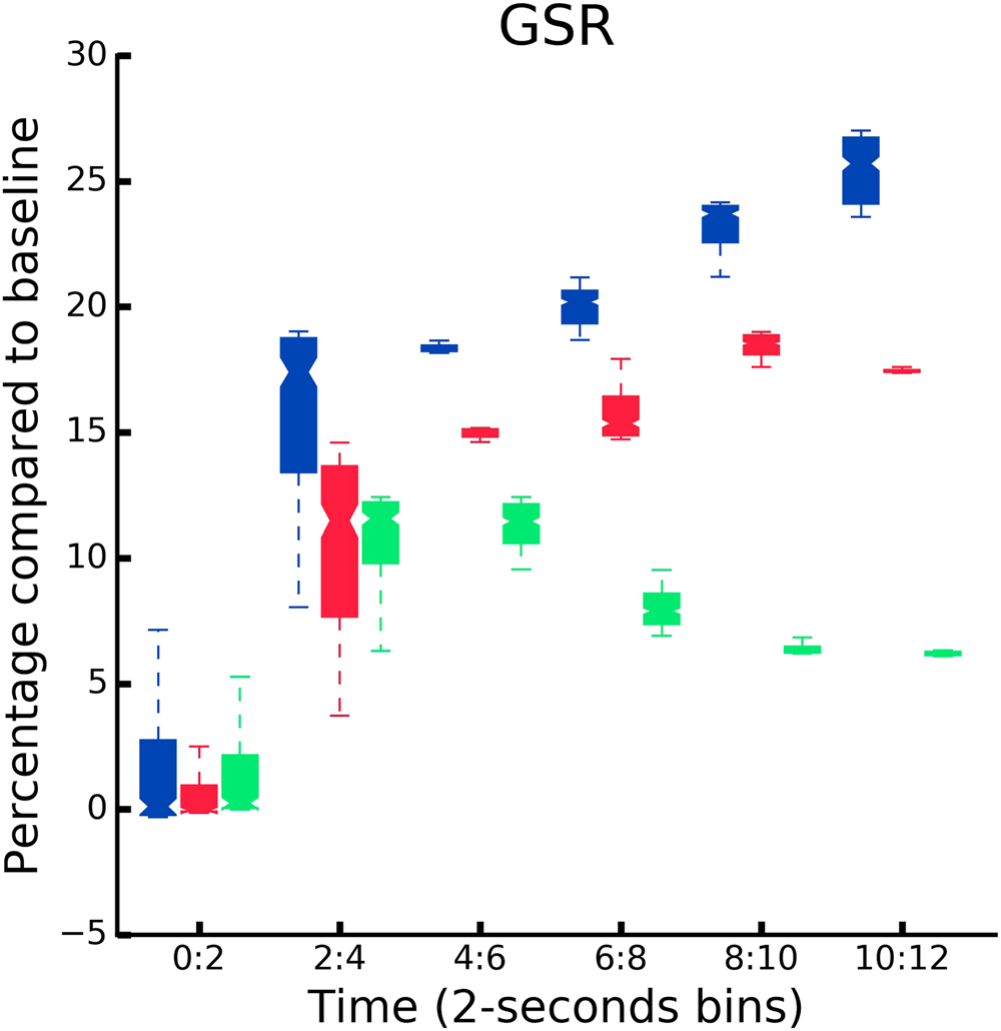
GSR responses per condition binned in 2.5-seconds time windows. The first bin (0–2 seconds) represents the latency of the GSR response following the threat (*time* = 0).

We stored and analyzed the GSR (Fig. 4) as a quantitative measure of ANS to further analyze differences in the induced feeling of ownership across the three experimental conditions. Prior to the data analysis, we calculated the mean GSR, as an integral of the curve in a time window of 12 sec, together with its associated standard deviation (SD) for every condition, and excluded three participants whose mean response was 2.5 SDs higher or lower than the mean of the group (2 participants whose signal was higher in C and IH, and 1 participant whose signal was lower in IV) (Fig. 5). As hypothesized, we found significant differences in the GSR data between the three conditions (*KW*, *H*(2,32) = 1256.5, *p* = 0.001). A Mann-Whitney follow-up test indicated that post-threat event mean GSR responses were significantly higher in C condition (*Mdn* = 13.64) than in IH (*Mdn* = 3.51, *U* = 229265, *p* < 0.001) and in IV (*Mdn* = 7.24, *U* = 328404, *p* < 0.001) conditions. Finally, we observed a significant difference between the control conditions IV and IH (*U* = 291783, *p* < 0.001). The GSR outcome further validates the results from the self reports suggesting the highest ownership in condition C.

### Performance

**Figure 6:**
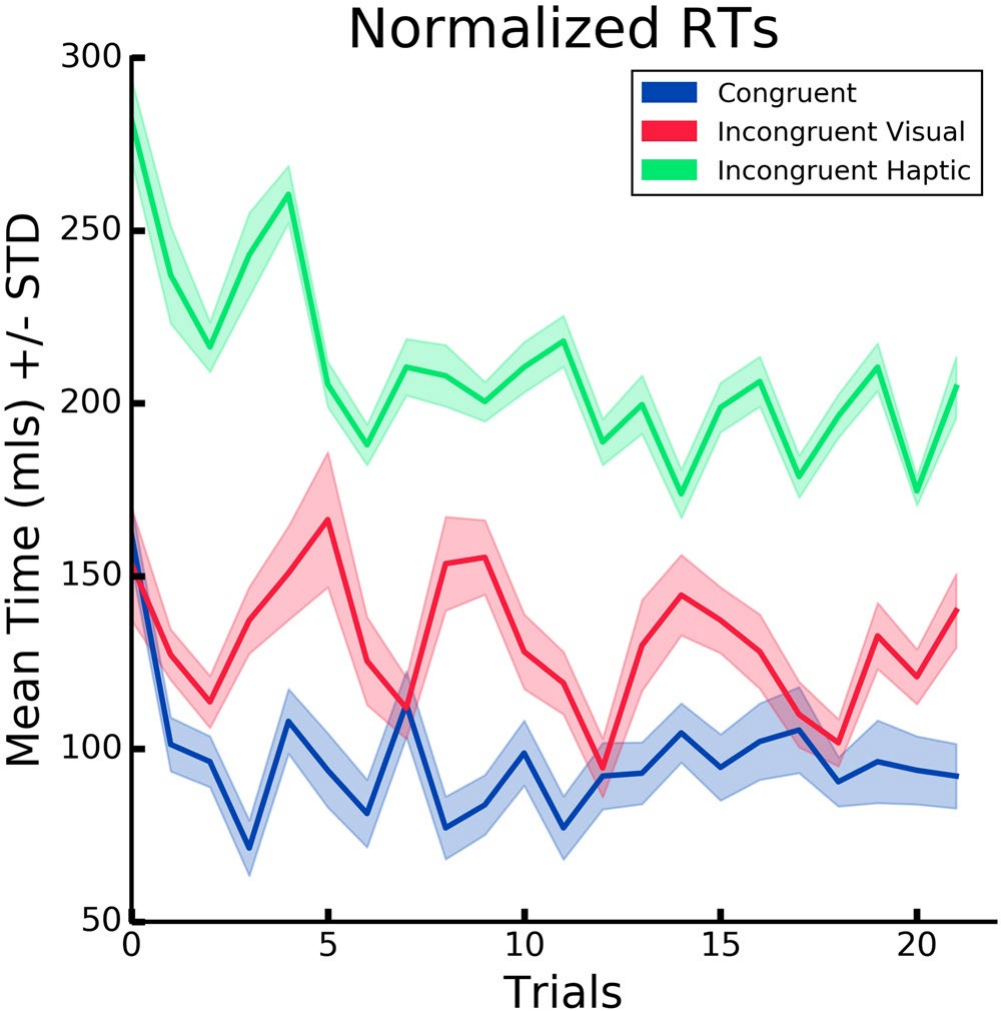
Response Times. Normalized mean of the response times for all the participants, defined as the intervals between the start of the stroking and subjects’ response, over time, per condition.

**Figure 7:**
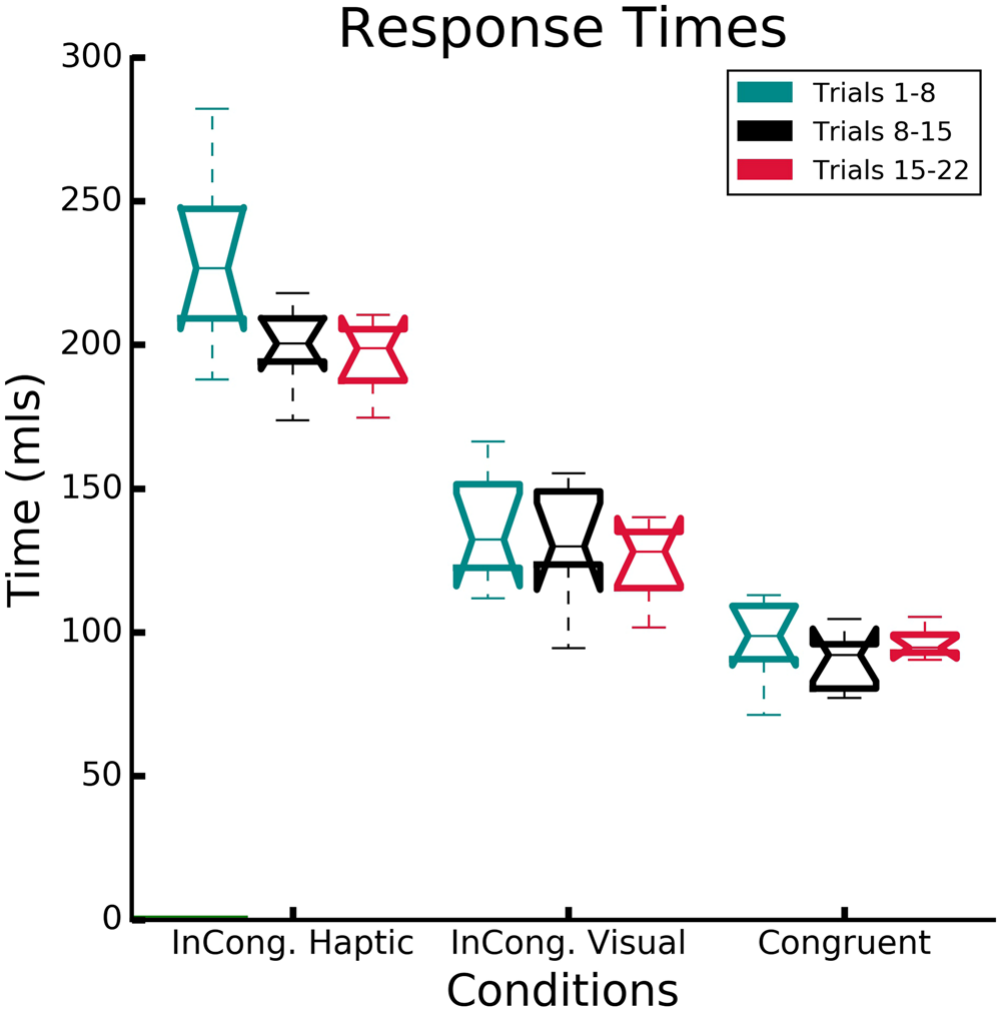
Normalized RTs responses for the three conditions binned in windows of 7 trials (1–8: early trials, 8–15: middle trials, 15–22: late trials).

Reaction times served as the performance measure in the proposed task. Medians per condition prior to normalization are: C (*Mdn* = 70.0), IV (*Mdn* = 105.0) and IH (*Mdn* = 200). The RTs measured in the first block (“spheres”) served to calculate the baseline (i.e. inter-subjects psychophysical differences) for every participant, which was subtracted from the intervention block for the performance analysis. As expected, in this block, no differences in RTs were found between the three conditions (*KW*, *H*(2,36) = 3.3, *p* = 0.19). In the intervention block, the reaction times served as a measure of motor performance in the proposed task. We observed a significant difference between the three conditions (*KW*, *H*(2,36) = 896.9, *p* < 0.001) (Fig. 6). A Mann-Whitney test further indicated that the RTs were significantly lower in condition C (*Mdn* = 94.16) than in both condition IV (*Mdn* = 129.0), (*U* = 480559.0, *p* < 0.001), and in IH (*Mdn* = 205.83), (*U* = 216680.5, *p* < 0.001). Additionally, we found that the RTs were significantly higher in the IH than in IV (*U* = 259008.5, *p* < 0.001). In condition IH, we found a significant difference between first, middle and last trials (*KW*, *H*(2,12) = 6.72, *p* = 0.034) (Fig. 7). No such differences were found in C (*KW*, *H*(2,12) = 2.34, *p* = 0.03) nor IV (*KW*, *H*(2,12) = 1.04, *p* = 0.51) (Fig. 7). We observe that the congruency of visuotactile stimuli modulated motor responses. We further report a difference in RTs between IV and IH possibly due to sensory predominance of vision over touch.

Finally, we computed the error responses in every condition for the experimental block including false positives (i.e. motor response provided given a stimulus to a finger different than the index finger) and anticipatory responses. For all the subjects and all the trials we report 4 errors in C, 3 in IV and 5 in IH. No significant difference was found in the errors’ RTs between the three conditions (*KW*, *H*(2,36) = 0.85, *p* = 0.65). Finally, we report no anticipatory responses in neither of the three conditions.

### Correlation Analysis

**Figure 8:**
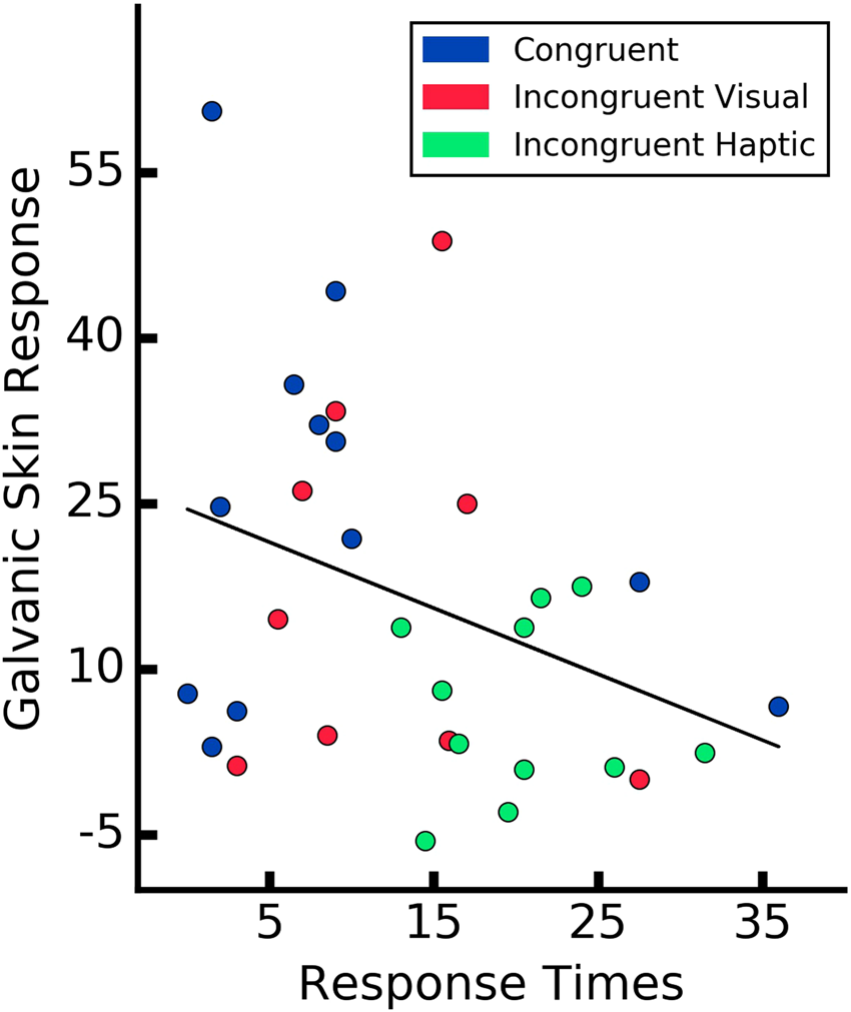
Correlation between the performance measure (mean RTs from the last five trials for each participant) and post-threatening galvanic skin response signal (mean GSR signal after the first peak). Every data point represents a participant.

**Figure 9:**
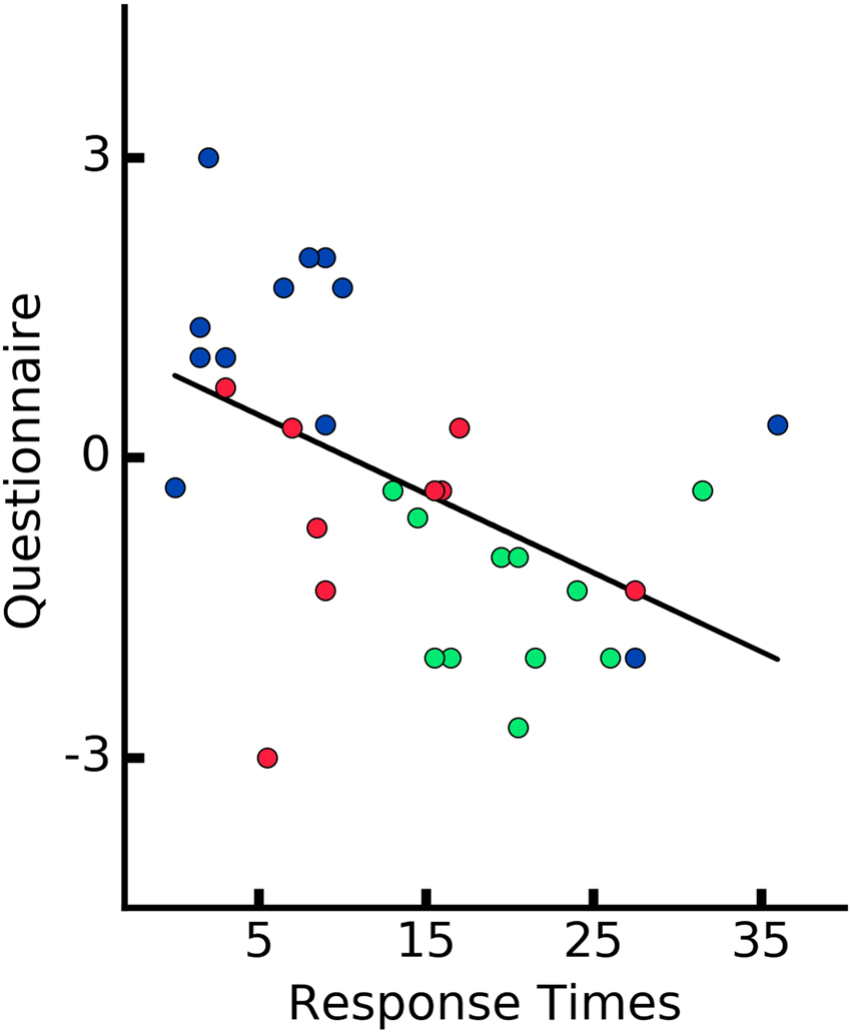
Correlation between the motor performance and self-reports (mean of the three ownership questionnaire for each participant).

**Figure 10:**
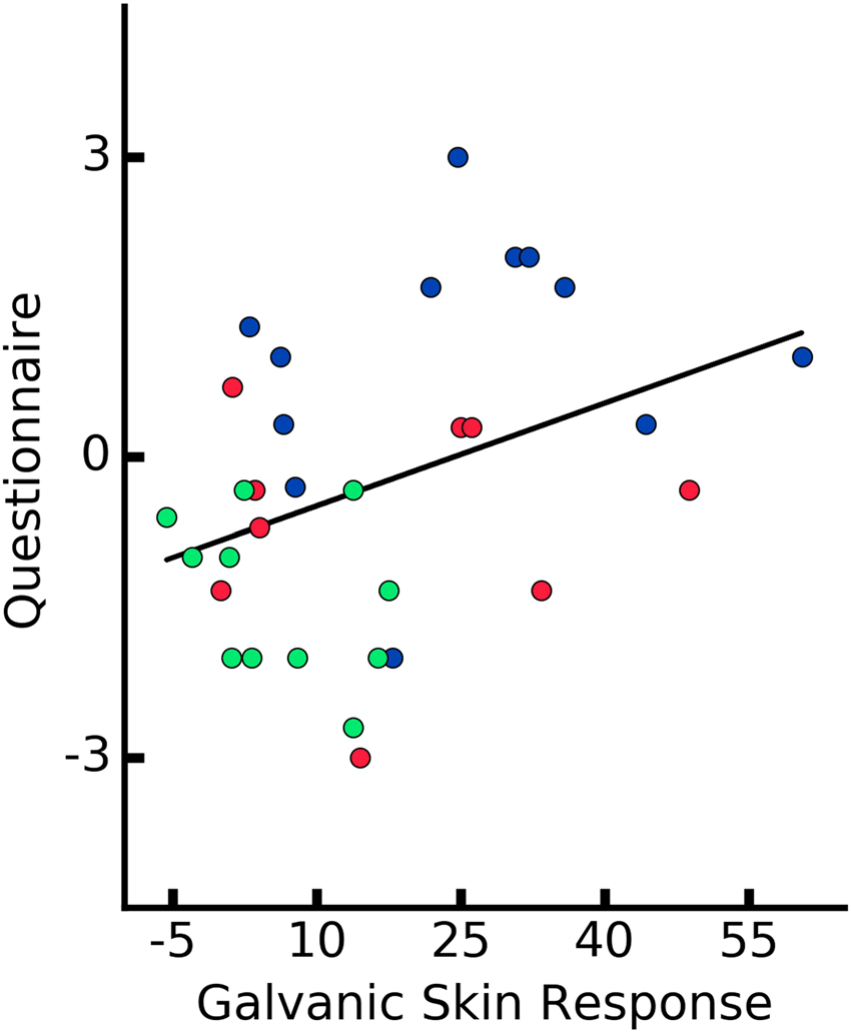
Correlation between the post-threatening galvanic skin response signal and self-reports. Every data point represents a participant.

A Pearson product-moment correlation coefficient was computed to assess the relationship between the ownership measures and performance on the motor task. For the analysis, we computed the mean GSR from the last five seconds post-threat, mean RT of the last 5 trials, and the mean score from the Ownership Questions for each participant in every condition, respectively. We report a significant negative correlations between post-threat GSR responses and the RTs (*r* = −0.35, *p* = 0.04) (Fig. 8) as well as the ownership questionnaire outcome and the RTs (*r* = −0.5, *p* = 0.003) (Fig. 10). Finally, the linear relationship was computed between the post-threat GSR responses and the ownership questionnaire. We observed a positive correlation between the two measures (*r* = 0.37, *p* = 0.03) (Fig. 9). This result confirms the consistency of the three different dimensions of ownership measure (i.e. behavioral, conscious report and physiological reaction). In addition, this results might suggest that the feeling of ownership can have different levels on a continuous scale rather than being a binary state.

## Conclusions

The goal of the present study was to investigate whether experimentally induced ownership can result in a modulation of motor performance in the proposed sensorimotor task. We hypothesized that the congruent, spatiotemporal pattern of visuotactile stimulation will account for higher feeling of ownership of a virtual limb and consequently enhanced performance in C as compared to the control conditions. To test this hypothesis we adopted the traditional RHI paradigm in a VR setting. The visual feedback of the active touch was manipulated across three conditions where we varied the congruence of inputs and the choice modality.

Results from self-reports suggest that the participants experienced touch in the location of the virtual hand when tactile and visual stimuli were delivered congruently. As expected, the scores in C were significantly different from both IV and IH conditions, where the stimulation was incongruent. To further assess the feeling of ownership and validate the data collected through self-reports, at the end of every experiment, we introduced a virtual threat and computed the GSR responses as an objective measure of autonomic, physiological arousal. We expected that the threatening event would provoke changes in GSR in all the three conditions, but that the subjects experiencing congruent visuotactile feedback would present higher responses due to the enhanced assimilation of the virtual hand into the body representation. Indeed, in C participants showed significantly higher GSR responses than in both IV and IH. Interestingly, we also observe statistically higher GSR responses in the IV than IH condition. Similar significant differences in performance (i.e RTs) were observed between the three conditions such that participants in C responded the fastest, and those in IH the slowest.

To investigate the relation between the degree of ownership, perceptual decision making and motor performance in the proposed task, we performed correlation analyses. We found that both subjective (i.e. self-reports) and objective (i.e. GSR) measures of ownership were significantly correlated which supports the use of present method to measure ownership. Secondly, we report significant correlations between both ownership measures and performance on the motor task. Interestingly, although both in IV and IH conditions the pattern of visuotactile feedback was incongruent and no ownership was expected, in the IV the performance and the GSR were higher than in IH while the measures were correlated. This might suggest that the attended modalities weighted ownership differently, such that despite the congruency of the feedback, when vision was attended to elicit motor action (i.e. IV) the ownership was higher than when the tactile stimuli was attended.

Overall, the reported results are consistent with previous studies within the framework of multimodal processing^10,15,23^ showing that the degree of ownership towards an artificial or, as in our case, virtual hand is associated with crossmodal processes of visuotactile stimulation, which can be measured both subjectively and objectively. Moreover, differences in response latencies between C and IV support that ownership has a modulatory effect on perceptual decision-making processes, which are coupled to behavior (i.e. RTs), physiological processing (i.e. GSR) and conscious perception (self-reports). Finally, our data suggests that ownership might be manipulated not only by the congruence of visuotactile inputs but also by the weight of the sensory stimuli that is being attended.

## Discussion

Accomplishment of even simple behavioral goals requires planning, execution, and complex coordination of movements involving different parts of the body^1,2^. Thus, in order to successfully interact within the external world, the brain needs to continuously process the information about the body and the surroundings so that it can adjust its internal model^17^ to the environment. The present results, in particular differences in performance between C and IV conditions, can be interpreted in terms of functional role of ownership and are consistent with previous literature in the domain of motor control^17,56^ supporting the idea that body ownership acts as an internal, dynamic model^57,58^ where bodily properties driven by multisensory integration can modulate motor performance. During the experiment, the participants had to make real-time decisions on whether to execute a particular motor action and press the button or not, depending on sensory inputs. When the visuotactile inputs were delivered congruently, such as in C, the amount of sensory information reduced perceptual ambiguity which triggered according motor response faster than when the incongruent stimuli were provided^59^. These differences in performance between C, IV and IH seem in line with the Bayesian principles of multimodal integration for decision-making mechanisms^19,59–61^. Alternatively, as had previous studies found^29^, this result may also indicate that congruent combination of information from different sensory modalities (i.e. vision and touch) facilitated the ability to recognize specific sensory stimuli resulting in faster perceptual discrimination and decision-making independent of ownership. Furthermore, during the experimental block subjects could learn to plan their responses differently when facing two asynchronous conditions IV and IH. We exclude, however, that the RTs might have been affected purely by the sensory congruency or expectations, since this effect would not lead to correlations between RTs and measures which did not directly depend on motor planning and motor control (i.e. GSR or self-reports). We believe that the present results highlight the role of ownership in the context of motor actions. We further observe, however, that the visuocactile integration also affected both the autonomous responses, measured through GSR and the conscious perception, measured through self-reports. This might suggest further that multisensory (i.e. visuotactile) integration has multiple dimensions including behavioral^10,18^ (i.e. motor), physiological^44^ and conscious^11,15,25,62^, which is supported by the correlation analysis between the discussed measures.

On the other hand, we observed significant differences in performance between IV and IH. Since the two conditions involved different sensory modalities to elicit motor response (i.e. visual or tactile), the reported result could be possibly explained by differences in terms of tactile and visual processing^29^. Given perceptual dominance of vision over touch, and coherent with literature^47,48^, in the IV the motor responses were faster than in IH condition. Interestingly, however, we further observe a significant difference in GSR responses between IV and IH conditions such that the GSR in the IV was significantly higher than in IH. We elaborated on this result by showing its relationship with the two other measures used in the study: autonomous physiological response and self reports. The reported correlations emerging from this analysis support the hypothesis that significant changes in reaction times across conditions could be effectively due to a modulation of the body representation. Furthermore, the high accuracy in motor performance, equally distributed in every condition, suggests that the difference in reaction times could be due to the same process of bodily representation affecting the subjective feeling of ownership. Since the visuotactile stimuli in IV and IH were incongruent, the feeling of ownership in IV should not have been modulated by sensory integration. Instead, we propose that the feeling of ownership in IV might have been modulated through the weight of the attended modality^29^, such that despite the congruency of the feedback, the ownership is higher when visual stimuli are attended to elicit motor action, than when the tactile stimuli are attended.

From the neuroscientific perspective, present results might be explained in terms of the anatomical coupling of brain structures underlying ownership and motor control. Clinical-pathological studies^6,32,30^ suggest that disturbances in attitudes towards body ownership tend to overlap with disorders in motor control, pointing to lesions in parietal and ventral premotor cortices, TPJ and the insula. The same set of brain areas have been identified in experimental studies investigating the multisensory nature of body ownership, using fMRI or PET^6,21,12,30^. Different performance outcomes presented here, seem coherent with this literature suggesting that perceptuomotor abilities result from multisensory integration mechanisms, such as in C condition. These mechanisms generate a coherent reference model and reduce perceptual ambiguity in the moment of decision-making enhancing the motor response. Such motor behaviour can be modulated by providing incongruent spatiotemporal sensory cues with varying weights^29^, as in IV or IH.

One of the critical aspects of the present study was that the participants were to provide motor responses using the left hand given sensory cues provided to the right hand with the induced ownership. Accordingly, we show that the degree of induced ownership indeed modulated the response times of the motor commands in the left hand. This result supports the theory that the coherence of the body is generated through agency (i.e. an accurate prediction and evaluation of the “reafference”) and leads to the reorganization and maintenance of body representations possibly located in the right insular cortex and the frontoparietal circuitry, the neural territories associated with the subjective experience of ownership^12^. In their study, Tsakiris and colleagues^63^, show that the integration of visuotactile stimulation induces body ownership locally, in a fragmented manner. They further propose, however, a secondary mechanism, possibly a generalization of the visuotactile associations, which accounts for perceiving the body as a coherent entity. Our results seem in coherence with this hypothesis suggesting that agency might play a role in the proposed secondary mechanism.

Applications of the presented paradigm might have relevance in fields such as motor rehabilitation. Acquired brain lesions including stroke often result in ownership disorders (i.e. anosognosia) and hemiparesis, which impair motor functions of upper extremities^35,37–39^. Recently, a number of studies examined the functionality of virtual reality based rehabilitation systems that aim at post stroke motor recovery of upper extremities^64–67^. Some of these setups are designed so that a motion sensor continuously tracks the user’s arms, and the movements are projected into the virtual scenario from a first person’s perspective. The underlying hypothesis for this rehabilitation research is that sensorimotor contingencies build up through experiential learning, and thus follow the statistics of the multimodal inputs that are exposed to the brain triggering plasticity which may lead to recovery^68,69^. Indeed, several studies show promising results^66,70–72^; however, none of them has explicitly addressed the question of whether inducing ownership towards the virtual effector might be beneficial for the rehabilitation purposes by reinforcing acquired sensorimotor contingencies and subsequently modulating arm use and motor performance. Further clinical studies will be conducted to evaluate whether the present method applies to hemiparetic stroke patients and whether induced ownership using virtual reality may influence recovery processes.
