# Author Correction: Visuotactile integration modulates motor performance in a perceptual decision-making task

**DOI:** 10.1038/s41598-017-15016-1

**Published:** 2017-11-10

**Authors:** Klaudia Grechuta, Jelena Guga, Giovanni Maffei, Belén Rubio Ballester, Paul F. M. J. Verschure

**Affiliations:** 1 Pompeu Fabra University, Laboratory of Synthetic Perceptive, Emotive and Cognitive Systems, Center of Autonomous Systems and Neurorobotics (SPECS), Barcelona, 08-018 Spain; 2 University of West Bohemia, New Technologies Research Center, Pilsen, 306-14 Czech Republic; 3 ICREA - Institució Catalana de Recerca i Estudis Avançats, Barcelona, 08010 Spain

*Scientific Reports* **7**:3333; doi:10.1038/s41598-017-03488-0; Article published online 13 June 2017

In the original version of this Article, Paul F. M. J. Verschure and Belen Rubio Ballester were incorrectly affiliated with ‘University of West Bohemia, New Technologies Research Center, Pilsen, 306-14, Czech Republic.’ The correct affiliations for Paul F. M. J. Verschure are listed below.

Pompeu Fabra University, Laboratory of Synthetic Perceptive, Emotive and Cognitive Systems, Center of Autonomous Systems and Neurorobotics (SPECS), Barcelona, 08¬018 Spain

ICREA - Institució Catalana de Recerca i Estudis Avançats, Barcelona, 08010 Spain

The correct affiliation for Belen Rubio Ballester is listed below.

Pompeu Fabra University, Laboratory of Synthetic Perceptive, Emotive and Cognitive Systems, Center of Autonomous Systems and Neurorobotics (SPECS), Barcelona, 08¬018 Spain

Additionally, this Article contained a typographical error in the spelling of the author Belen Rubio Ballester, which was incorrectly given as Belen Ballester Rubio.

These errors have now been corrected in the PDF and HTML versions of the Article.

